# All-Hydrocarbon Low-Dielectric Loss Benzocyclobutene-Encapsulated Photoresist with High Pattern Resolution

**DOI:** 10.1021/acsomega.4c10940

**Published:** 2025-04-12

**Authors:** Hanlin Du, Hongyan Xia, Yun Tang, Ke Cao, Jiajun Ma, Junxiao Yang

**Affiliations:** † School of Materials and Chemistry and State Key Laboratory of Environmentally-Friendly Energy Materials, Southwest University of Science and Technology, Mianyang 621010, China; ‡ Department of Applied Sciences, Northumbria University, Newcastle Upon Tyne NE1 8ST, U.K.; § School of Materials and Construction, Mianyang Polytechnic, Mianyang 621000, China

## Abstract

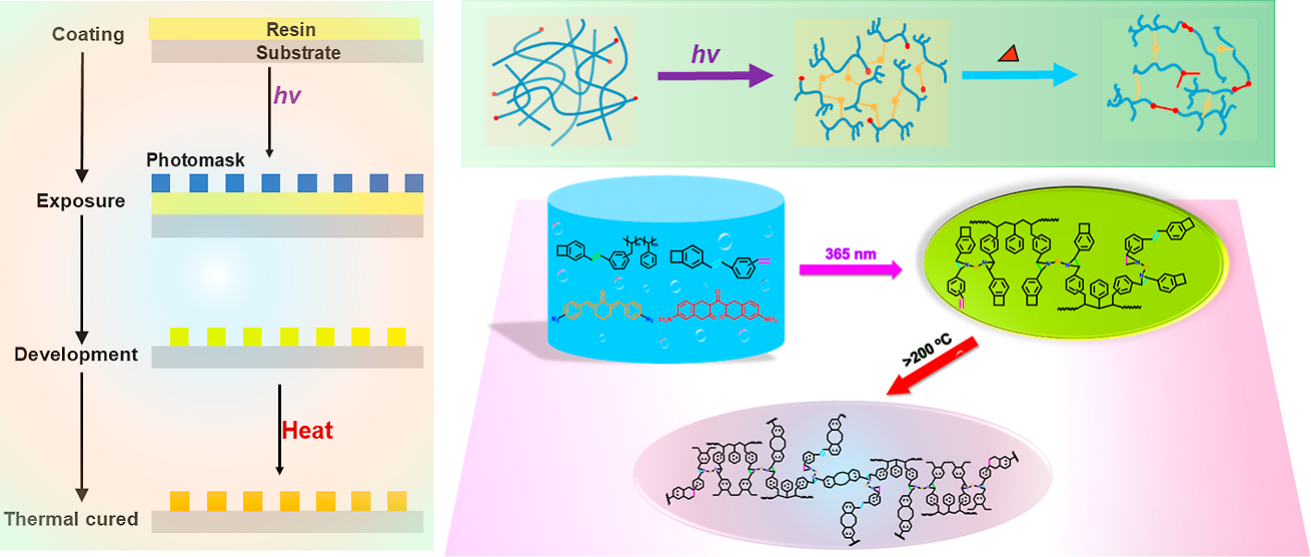

UV-curable resins with a low dielectric constant can be processed or patterned to form required shapes, making them highly applicable to special fields. Unlike conventional photoresists limited by the polarization effect due to highly polar bonds, an all-hydrocarbon-type low-dielectric photoresist was designed and synthesized with excellent performance. Based on previous works, the film-forming resin poly 1-(4-vinylphenyl)-2-(4-benzocyclobutenyl)ethylene-styrene (P-DVB-St) was prepared by introducing styrene (St) into the 1-(4-vinylphenyl)-2-(4-benzocyclobutenyl)ethene (DVB) backbone via anionic polymerization, and the photoresist properties were improved by adjusting the cross-linking density of the polymer. The introduction of styrene improved the mechanical properties while maintaining the photolithographic patterning properties of the photoresist. Since the resin has a dual UV/thermal-cured structure, it has better thermal stability (*T*_5%_ = 401 °C), lower dielectric constant (2.62 at 10 MHz) and dielectric loss (1.7 × 10^–3^), and better photolithographic patterning (the graphic resolution is 5 μm).

## 1 Introduction

The microelectronics industry is advancing rapidly, with 5G technology, the next-generation 6G technology and artificial intelligence developing quickly. This has led to the miniaturization of electronic devices and integrated circuits (ICs), making them smaller, faster, and more dense. 5G technology, using high-frequency devices, is expected to enable high-speed wireless communication.^1,2^ Meanwhile, fan-out wafer level packaging (FO-WLP) has reduced the package size and cost and is a significant semiconductor technology.^3^ The redistribution layer in FO-WLP requires dielectric materials with a low dielectric constant (*D*_k_ < 2.5) and loss factor (*D*_f_ < 0.002) to accommodate more metal wires at narrower spacing.^4,5^ Furthermore, future electronic devices and antennas may use irregular components with unique porous structures, requiring resins that are easy to process and can be patterned using new technologies like UV-NIL.^6,7^

In recent years, UV-cured technology has gained popularity due to its advantages of high efficiency, low cost, and environmental friendliness.^8−10^ By adjusting the formulations and process parameters, it can meet the needs of traditional or emerging applications. The technology uses photosensitive oligomer resin as a substrate, with different lithography systems to achieve three-dimensional all-round, multidimensional applications. The physical and chemical properties of the matrix resin significantly affect the quality of the cured resin.^11^

Photoresist is a crucial raw material in the production of ICs. During the IC manufacturing process, the photochemical reaction in the exposed area causes the exposed and nonexposed areas to have different solubilities in the developer. After the pattern is transferred to the substrate material, the photolithographic pattern can be removed or become the lithographic insulating pattern of the IC, forming the required LIPs for the device.^12−15^ Organic polymers exhibit greater advantages in terms of electrical and film-forming properties, as well as controllability of properties, due to their structural characteristics. Up until now, high-performance low-dielectric polymer materials such as PTFE,^16^ PI,^17^ SILK,^18^ and DVSBCB^19^ have been developed. Jiang et al.^20^ synthesized an acrylate-based fluorinated hyperbranched photosensitive polyaryletherketone (hb-P6FAEK-Ace) as a prepolymer. With different bifunctional aliphatic active diluents and 4-acryloyl morpholine, they prepared UV-cured films. Notably, the CF-DCDDA film exhibited a low dielectric constant of 2.87 at 20 GHz. Zhang et al.^21^ utilized photocurable fluorinated poly(phthalazinone ether) (FSt-FPPE) as a prepolymer with acrylic diluents to create UV-curable inks named FST/DPGs. After curing, the films showed excellent properties, with the 20% DPG film achieving a dielectric constant of 2.75 at 20 GHz. Yang et al.^22^ introduced three selected difunctional acid chain extenders into 1,6-naphthalene diglycidyl ether (NDE) to synthesize a series of naphthalene-type photocurable enhancement resins. The optimized UV-cured S-NDE thin film demonstrated a desirable dielectric constant of 2.2 at 10–10^7^ Hz. Liu et al.^23^ reacted cholic acid with epoxy resin and further modified it with glycidyl methacrylate to obtain a novel UV-light-sensitive solid resist resin (ECTG). The ECTG3 sample had a low dielectric constant of 2.51 at 10 MHz.

However, most of these polymers have some common defects: (1) they have a low dielectric constant but cannot guarantee accuracy and resolution of patterning because they are nonphotosensitive materials;^24^ (2) their thermal stability is usually lower than that of inorganic materials.^25^ As the dielectric insulating layer between metal wires and unit chips, the film needs to have a low dielectric constant (*D*_k_) and dielectric loss (*D*_f_) and achieve a higher resolution patterning^26^ to effectively insulate metal wires.^27^ In addition, it must have a low coefficient of thermal expansion (CTE), high mechanical properties and bonding strength, a high glass-transition temperature (Tg), and sufficient flexibility and humidity resistance to ensure reliability.^28,29^

The film-forming resin based on benzocyclobutene has the advantages of low dielectric constant, low dielectric loss, low moisture absorption, high thermal stability, and so forth. In this paper, an all-hydrocarbon benzocyclobutene film-forming resin was designed and synthesized from the viewpoint of the influence of structure on performance, and styrene was introduced into the main chain of the polymer through anionic polymerization reaction. The thermodynamic and electrical properties were controllably adjusted by reducing the cross-link density of the polymer. The resin has photoactive groups, and the compounded photosensitive system with azidocyclohexanone (BAC) can realize UV lithography patterning, with the pattern resolution reaching 5 μm, and the benzocyclobutene groups act as thermal cross-linking groups to give the polymers excellent mechanical and dielectric properties after thermal curing. Finally, this paper presents a comprehensive analysis of the UV/thermal-cured kinetics of the photoresist, making it a potentially applicable encapsulated photoresist.

## 2 Experimental Section

### 2.1 Materials

1-(4-Vinylphenyl)-2-(4-benzocyclobutenyl)ethene is synthesized according to literature methods^29^. Styrene is purchased from Chengdu Cologne Chemical Industry and needs to be revaporized before being used. 2,6-Bis(4-azidobenzylidene)cyclohexanone (BAC) and 3,3′-carbonylbis(7-diethylaminocoumarin) (dye) were purchased from TCI (Shanghai) Development Co., Ltd. Tetrahydrofuran (THF) and *n*-butyllithium (2.5 M in hexane) were purchased from Aladdin. Tetrahydrofuran (THF) was dried before use. Dipropylene glycol dimethyl ether, mesitylene, and cyclopentanone were supplied as solvents by Aladdin Chemical Industry Co., Ltd. Petroleum ether (PE) was supplied as a solvent by Mianyang Rongsheng Chemical Industry Co., Ltd.

### 2.2 Characterization

The molecular structure was identified at room temperature using a Bruker AVANCE-600 nuclear magnetic resonance (NMR) spectrometer, with deuterated chloroform (CDCl_3_) and tetramethylsilane as the solvents. The vibrational modes in the samples were determined by Fourier transform infrared (FTIR) spectroscopy within the range of 400–4000 cm^–1^, employing a Nicolet-7500 IR spectrometer. The sample membranes were prepared by solution calendering onto a KBr support sheet. The thermal stability properties of the UV/thermal-cured resins were evaluated by thermogravimetric analysis (TGA) using an SDT-2960 analyzer (TA Instruments, USA). Here, the temperature was increased from ambient temperature to 800 °C at a rate of 20 °C/min under a nitrogen atmosphere, to obtain the TGA curves. The thermal properties of the UV/thermal-cured resins were further evaluated by differential scanning calorimetry (DSC) using a TA-Q2000 calorimeter (TA Instruments, USA). Again, the temperature was increased from ambient temperature to 800 °C at a rate of 20 °C/min under a nitrogen atmosphere, to obtain the DSC curves. The mechanical properties of the UV/thermal-cured resins were evaluated using a G200 nanoindentation instrument (KLA, USA). A strain rate of 0.2 s^–1^ was maintained while continuously increasing the load until the indenter reached a depth of 2000 nm on the sample’s surface, and then the maximum load was held for 10 s. Thermomechanical analysis (TMA) was performed to evaluate the thermomechanical properties and coefficient of thermal expansion using a Discovery TMA 450 analyzer (TA Instruments, USA), and the temperature was incrementally raised from 30 to 300 °C at a rate of 5 °C/min under a nitrogen atmosphere to generate the TMA curves.

Initially, the formulated photosensitive solution underwent UV illumination to achieve photo-cross-links. Subsequently, heat treatment facilitated thermal cross-linking, resulting in the formation of cast body. After surface polishing, the cross-linked resin’s thickness was measured using a micrometer. Electrodes were then created on the top and bottom surfaces of the cast body by applying silver paint using a brush. Material capacitance was quantified by measuring the impedance using an Agilent HR4294LCR precision meter over a frequency range of 0–10 MHz. The dielectric constant was calculated using the following formula
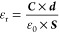
where *S* represents the area of the sample, *d* represents the thickness of the sample, *C* represents the capacitance, and ε_0_ represents the vacuum dielectric constant (8.854 × 10^–12^ F/m).

### 2.3 Synthesis of Poly 1-(4-vinylphenyl)-2-(4-benzocyclobutenyl)ethylene-styrene

A 25 mL dry single-necked flask was charged with DVB monomers (0.998 g, 4.3 mmol), and the system was evacuated and operated with nitrogen to ensure that it was free of water and oxygen. Subsequently, styrene (0.5 g, 4.3 mmol) and 10 mL of THF were added and fully stirred to dissolve. The flask was then transferred to a cryogenic bath at −78 °C and frozen for 1 h. The reaction was initiated by the addition of 0.5 mL of *n*-butyllithium, resulting in a change in solution color from pale yellow to purple-black. The reaction was terminated by the addition of 1 mL of methanol after 6 h. The light yellow transparent liquid obtained was slowly poured into 100 mL of anhydrous ethanol, resulting in the precipitation of a white solid. The white solid was filtered under vacuum, and the resulting sample was baked in a vacuum at 60 °C for 8 h, yielding 1.27 g of the desired product. The yield was 85%. Three copolymerized P-DVB-St resins were obtained by adjusting the monomer reaction feed ratio (DVB: St), as shown in Table 1.

**Table 1:** Polymerization of DVB and St with Different Conditions

sample	feedstock ratio (DVB: St)	wt % of monomer on THF	*n*-BuLi (mmol)	reaction condition	M_w_ (g/mol)	PDI	actual graft ratio
P1	3:7	14.4	1.25	–78 °C, 6 h	1.25 w	1.11	3:9
P2	5:5	14.4	1.25	–78 °C, 6 h	1.4 w	2.45	5:5
P3	7:3	14.4	1.25	–78 °C, 6 h	1.8 w	1.56	5:2

### 2.4 Preparation of Photoresists and Cured Samples

In a light-proof environment, P-DVB-St (0.3 g), photoinitiator BAC (0.015 g), co-initiator dye (0.003 g), monomer DVB (0.045 g), mesitylene (0.6 g/0.35 g), and cyclopentanone (0.6 g/0.35 g) were added to a 3 mL brown sample vial, ultrasonically dissolved, and microwell-filtrated to obtain photosensitive solutions (20%/30% solid content). Subsequently, the photosensitive polymer film (uncured) was prepared by spin-coating on a glass or Pt/Si substrate. Lithography patterning is performed using 365 nm UV-LED, and the developer is DS1100 (DME/PE = 70:30). The photolithography process parameters are shown in Table 2. Finally, the UV-cured film was vacuum-cured in an oven with the following warming procedure: 160 °C for 1 h, 180 °C for 1 h, 200 °C for 2 h, 215 °C for 2 h, 230 °C for 2 h, 215 °C for 1 h, 200 °C for 1 h, 180 °C for 1 h, and 160 °C for 1 h, followed by natural cooling down to room temperature, to obtain a high cross-linking density (UV/thermal dual-cured film).

**Table 2:** Photolithography Process Parameters

photolithography process		spin speed (rpm)	prebake (°C)	expose (mW/cm^2^)	immersion develop	postbake (°C)	thickness (μm)
process parameter	20 wt % of material in solution	3000	70	600 × 30 s	DS1100	80	1–2
	30 wt % of material in solution	2000	70	600 × 120 s	DS1100	80	6–7

The configured photosensitive solution was added to a light-safe cured tube, and the solvent was evaporated for several hours at 70 °C ambient temperature before further vacuum drying. The cured tube was placed under a UV light source for 15 min to completely cure the sample and then placed in a vacuum oven to raise the temperature according to the following procedure: 140 °C for 1 h, 160 °C for 1 h, 180 °C for 1 h, 210 °C for 2 h, 240 °C for 5 h, 200 °C for 1 h, 180 °C for 1 h, and 160 °C for 1 h, followed by natural cooling down to room temperature, to obtain a solid sample with a high cross-linking density.

## 3 Results and Discussion

### 3.1 Synthesis and Characterization of Polymers

**Figure 1:**
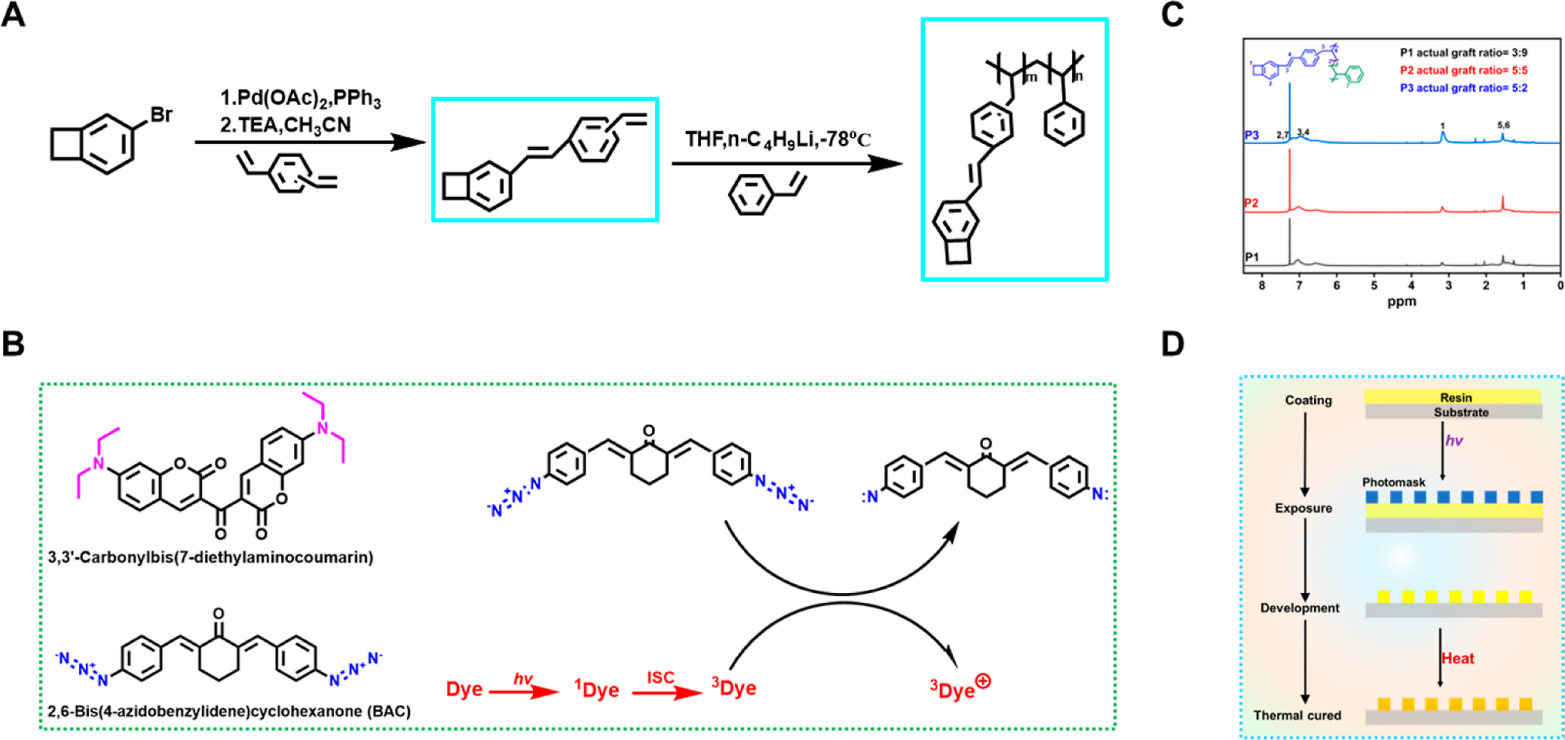
(A) Synthesis route of P-DVB-St; (B) structure of photoinitiators and free-radical generation mechanism of two-component photoinitiation systems; (C) ^1^H NMR spectrum of P-DVB-St; (D) patterning of the BCB photoresist and schematic of the thermal curing process.

P-DVB-St resins can be synthesized expeditiously and on a substantial scale through anionic polymerization. The molecular structure and molecular weight can be modified by modifying the reaction feed ratio and polymerization conditions. The synthesis path is shown in Figure 1A. Figure 1B is the photoinitiator system used in the photoresist. The excited state of coumarin initially undergoes an electron transfer with azidocyclohexanone. The excited state of azidocyclohexanone is in an unstable structural state, and the azide group undergoes homolytic cleavage to produce primary reactive species. These reactive species then bridge with oligomers to form polymers with a certain degree of cross-linking. Figure 1C illustrates the ^1^H NMR spectrum of the P-DVB-St resin. The chemical shifts observed at 0.8–2.1 ppm are indicative of the saturated carbon–hydrogen structure of the polymer. Additionally, the hydrogen proton peaks of the BCB four-membered ring are evident at 3.17 ppm. The chemical shifts at 6.5–7.3 ppm are attributed to the proton peaks of the benzene hydrogens. Notably, the double bond exhibits a shift toward the lower field, which can be attributed to the conjugation with the benzene ring. This observation is consistent with the presence of benzene ring hydrogens.^30^ According to the *Q*–e concept, the conjugation effect of DVB is stronger than that of St. Therefore, the *Q* value of DVB is larger, which is more conducive to anionic polymerization, and the copolymerization of the two is therefore inclined to be random copolymerization.^31^Figure 1D provides a schematic representation of the UV/thermal-cured process employed for the P-DVB-St resin. The photoresist is developed by UV exposure to form a photolithographic pattern with a specific cross-link density, Subsequently, the UV-curable resin is thermal-cured via the thermal ring-opening reaction of the BCB unit, thereby yielding the P-DVB-St resin. Refer to Figure S1 for the molecular weight spectra and Figure S2 for the film thickness curve. P-DVB-St resin purity is shown in Figure S3.

### 3.2 Photolithography Patterning Performance Properties of P-DVB-St Resin

**Figure 2:**
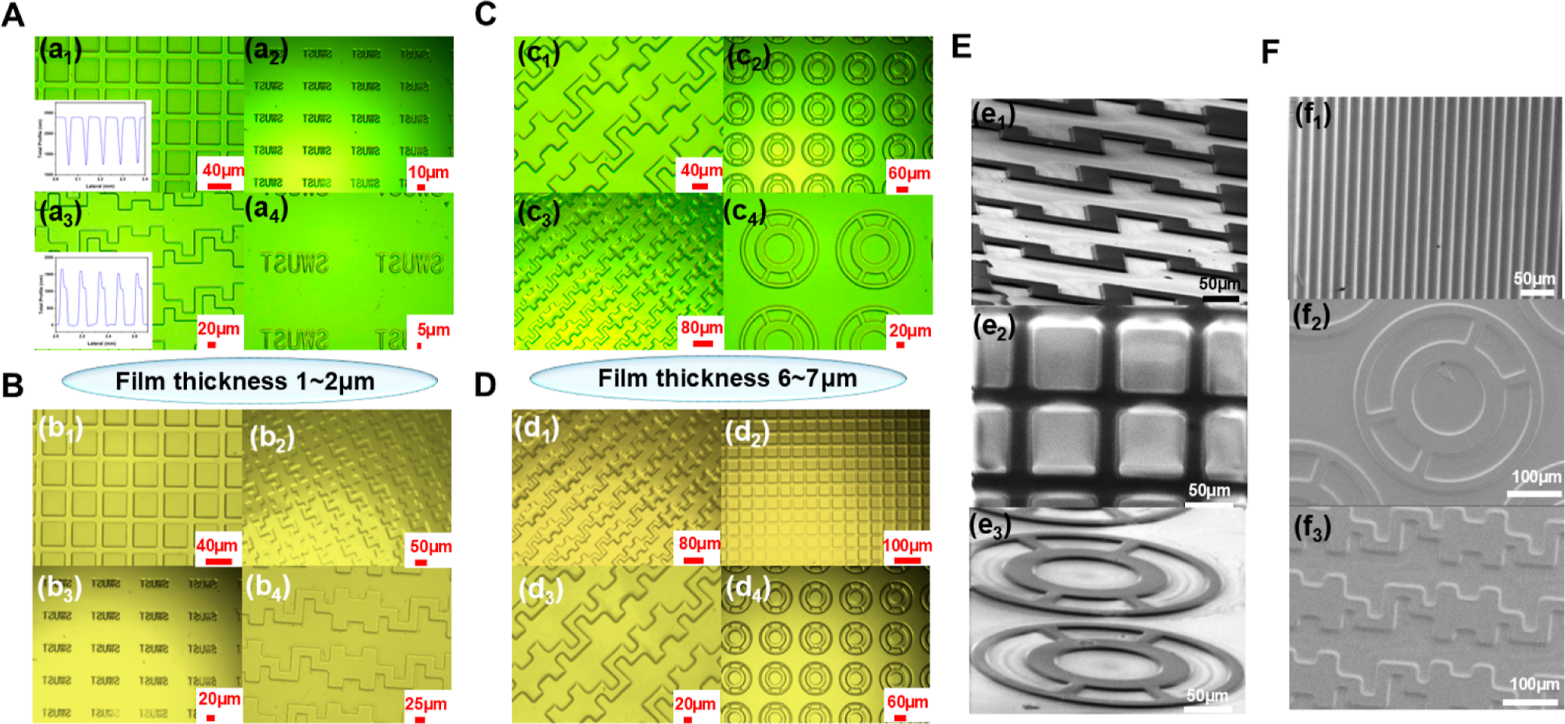
(A) 1–2 μm film photolithography pattern effect. (a1) Square with a side length of 40 μm and its corresponding step meter film thickness; the step gauge film thickness profile is shown in the lower left graph for (a1); (a2) “SWUST” letters with a line width of 5 μm; (a3) sawtooth patterns and their corresponding step meter film thicknesses for the middle connecting line of 20 μm. The step gauge film thickness profile is shown in the lower left graph for (a3); (a4) “SWUST” pattern under a high magnification. (B) Photomicrographs of UV/thermal-cured patterns. (C) 6–7 μm film photolithography pattern effect. (c2) Concentric rings with an outer ring line 10 μm wide, an inner ring line 20 μm wide, and a connecting line of 10 μm, and the rest of the pattern is consistent with (A). (D) Photomicrographs of UV/thermal-cured patterns. (E) SEM image of the UV-cured pattern. (F) SEM image of the UV/thermal-cured pattern. (f1) Lines with a width and spacing of 15 μm.

Photoactive low-dielectric materials can be directly patterned by photolithography, eliminating the step of removing sacrificial patterning materials and simplifying the packaging process. In this study, 365 nm UV lithography of two photosensitive films with varying film thicknesses was employed to examine the photolithographic patterning capabilities of the photoresist. The surface morphology of the UV/thermal-cured patterns was then analyzed by SEM. Figure 2A,B shows the light-cured and light/thermal-cured patterns of 1–2 μm photosensitive films, respectively, and Figure 2C,D shows the light-cured and UV/thermal-cured patterns of 6–7 μm photosensitive films, respectively. The UV-cured patterns have a high degree of reproduction, good edge steepness and straightness, no obvious deformation and adhesion, and they can also be well printed for complex patterns (such as “SWUST”), and the resolution of the patterns is up to 5 μm, with the edge roughness of 0.5 μm. The UV-cured pattern was thermal-cured at a high temperature without deformation of the graphic, and the film remained intact, exhibiting no signs of cracking or graying out. This suggests that the photoresist possesses excellent thermal stability. The photolithography patterns for commercial DVSBCB photoresists are shown in Figure S4.

Figure 2E is the SEM image of the UV-cured film, and there is a slight collapse on the surface of the pattern, which is caused by the indentation of the mask plate as well as the contraction of the UV-cured film. Figure 2F depicts the SEM image of the UV/thermal-cured film. The pattern is discernible, and the surface finish is high. This is attributed to the elevated temperature during the thermal-cured process, which induces the rearrangement of chain segments, repairs surface defects, and facilitates the formation of a cross-linked network. The latter phenomenon inhibits volume shrinkage during the curing process, thereby imparting the film with a high degree of flatness.

### 3.3 Dynamical Studies of UV-Cured P-DVB-St Resin

**Figure 3:**
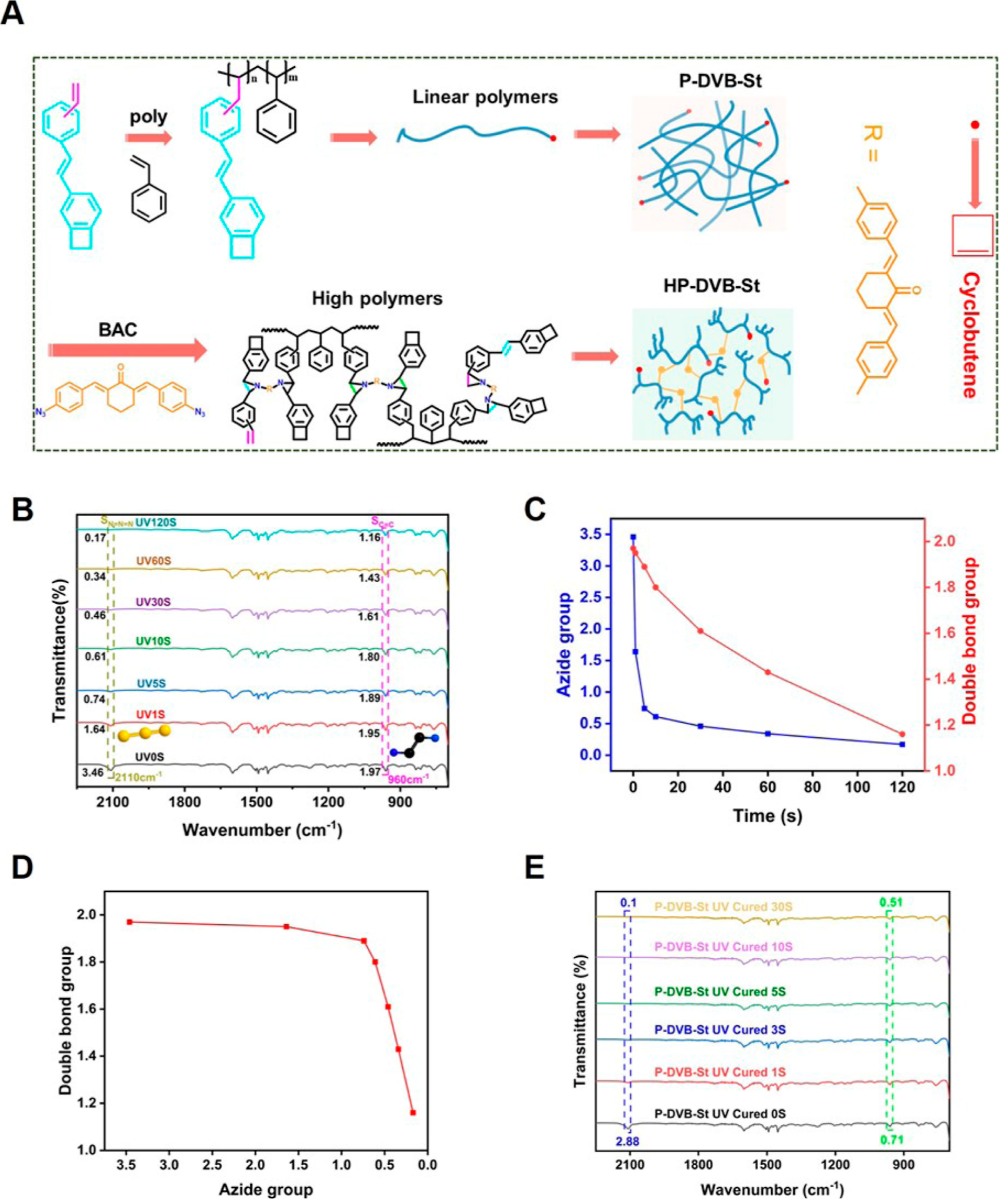
(A) UV-cured mechanism diagram of P-DVB-St. (B) UV-cured kinetic FTIR spectrum of P-DVB-St. (C) Changes in the content of azide groups and double-bonding groups with the exposure time. (D) Variation of double-bonding groups with the content of azide groups. (E) 1400 mW/cm^2^ UV-cured kinetic FTIR spectrum of P-DVB-St.

Figure 3A illustrates the photolithography mechanism of the P-DVB-St photoresist. The photoinitiator system produces azidocyclohexanone azocarbene through an electron transfer reaction under 365 nm UV exposure. This is followed by the formation of an azacarbene ternary ring, which results in the generation of an all-carbon–hydrogen polymer with a specific cross-linking density.^32−35^ This ultimately leads to a substantial reduction in the solubility of the polymer in the developer solution, thereby enabling the achievement of high-resolution imaging. The UV-cured kinetic FTIR spectrum of P-DVB-St is shown in Figure 3B, and the integral area of the N=N=N absorption band at 2110 cm^–1^ was 3.46 nm before being cured. The azocarbine peaks gradually weakened with the increase in the UV exposure time. The integral area of the N=N=N absorption band became 0.17 after being UV-cured for 120 s. At this time, the efficiency of photoinitiator conversion to azocarbene was 95.08%.

Figure 3C,D shows the changes in the content of azide groups and double-bonding groups with the exposure time, and the changes in the content of double-bonding groups with the azide groups, respectively. The azide group content and double-bond content were calculated by integrating the area of 2110 and 960 cm^–1^ by the FTIR spectrum. The phenomenon of self-acceleration is observed in the initial stages of the curing process, wherein the polymer exhibits extended kinetic chain segments and a precipitous surge in azocarbine-reactive species, which markedly facilitates polymer bridging. As the number of DVB groups attached to the backbone increases, the kinetic chain segments of the polymer backbone become shorter and more difficult to move. The three-dimensional mesh structure formed by curing further restricts the movement of the chain segments and azocarbine, eventually leading to a plateau in the reaction and the termination of polymerization. This is reflected in the curve as a “slow–fast–slow” trend.

In this study, the impact of light intensity on the light-cured reaction was also examined by elevating the light intensity from 600 to 1400 mW/cm^2^, as illustrated in Figure 3E. Following a 30 s exposure to light, the peak area of the azide group at 2110 cm^–1^ was reduced from 2.88 to 0.1, indicating a nitrogen carbene conversion of 96.52%. In comparison to the light intensity of 600 mW/cm^2^, the curing efficiency demonstrated a 400% increase. In addition, we also investigate the kinetics of UV curing at a light intensity of 150 mW/cm^2^, as shown in Figure S5. The UV–vis absorption spectrum of the photoresist is shown in Figure S6.

### 3.4 Dynamical Studies of UV/Thermal-Cured P-DVB-St Resin

**Figure 4:**
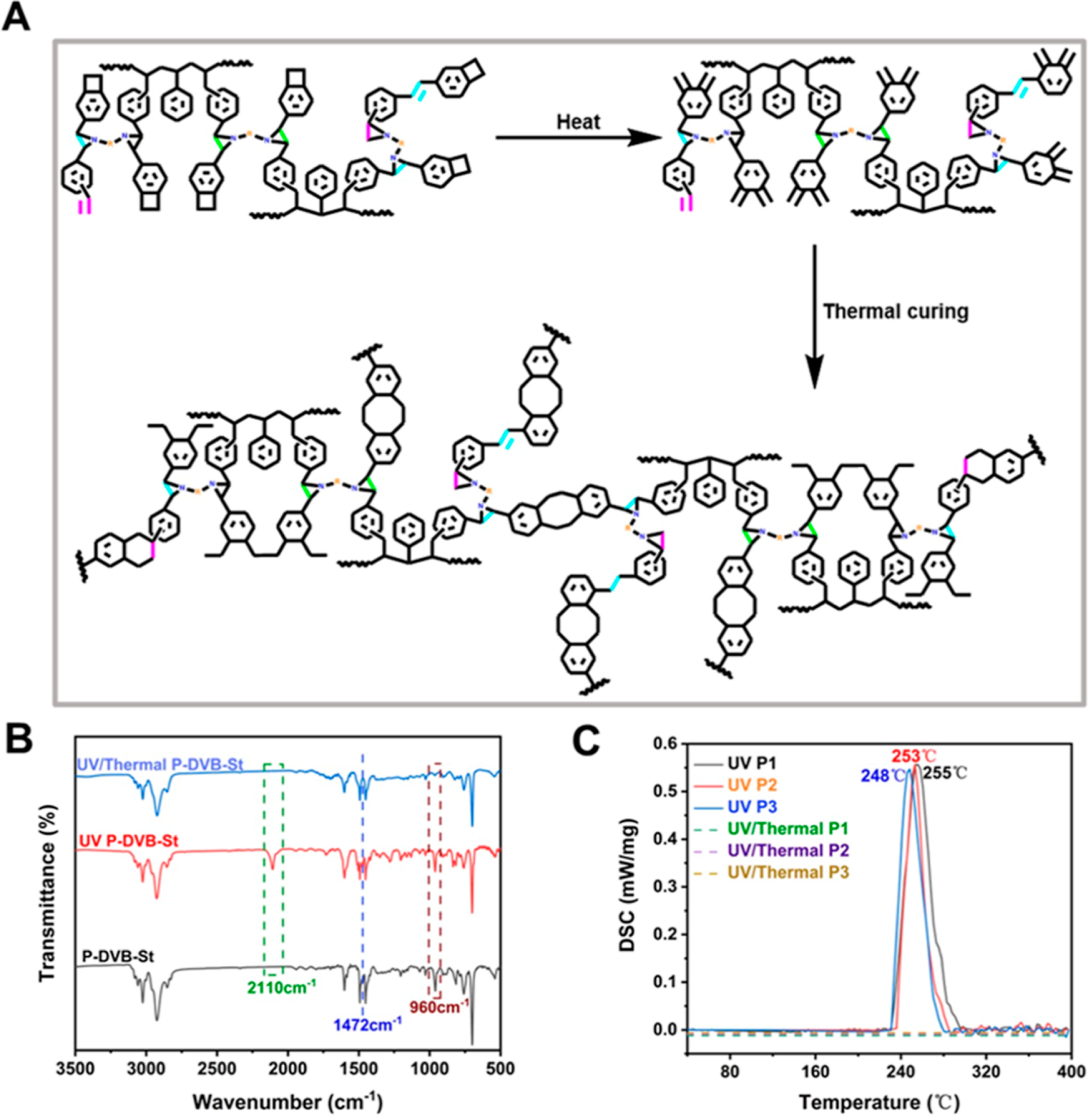
(A) Heat-cured mechanism diagram of P-DVB-St. (B) FTIR spectra of pure P-DVB-St resins, UV-cured P-DVB-St resins, and UV/thermal-cured P-DVB-St resins. (C) DSC curves of UV-cured P-DVB-St resins and UV/thermal-cured P-DVB-St resins.

The resin still has a high number of active cured sites (double bonds and BCB groups) after UV curing, so heat curing is used to activate these cross-linking sites and enhance the polymer properties. Figure 4A shows the heat-cured mechanism for UV-cured P-DVB-St resins. The benzocyclobutene opens the ring above 200 °C to generate the reactive intermediate *o*-dimethylenequinone, which undergoes a Diels–Alder reaction with the pro-dienophile or itself to generate a benzo-six-membered ring or a benzo-eight-membered ring to form a dense three-dimensional cross-linked network structure. The study characterized its thermal-cured process by infrared spectroscopy (Figure 4B). The vibrational peak of the benzotetrameric ring at 1472 cm^–1^ disappeared after cured and was at 960 cm^–1^ of the double-bond bending out of the trans face also basically disappeared, indicating that the double bond had been completely cross-linked. In contrast, the azide groups located at 2110 cm^–1^ that were not fully reacted away during the light-cured stage were also fully reacted after heat curing.

The thermal cross-linking cured process of polymer P-DVB-St was subjected to further investigation by DSC, as illustrated in Figure 4C. The resulting DSC curve exhibited a single exothermic peak, with a maximum exothermic peak temperature of approximately 250 °C. This peak is attributed to the exothermic peak of the ring-opening polymerization of four-membered BCB rings. The double bond is challenging to self-couple due to the site resistance effect and primarily undergoes a Diels–Alder cycloaddition reaction with the active intermediate *o*-dimethylenedioxyquinone subsequent to the BCB ring-opening reaction. Consequently, the exothermic peak of the double bond coincides with the exothermic peak of the ring-opening polymerization of the BCB moiety, This is also confirmed by the study through rheology (see Figure S7).

### 3.5 Composite Properties of UV/Thermal-Cured P-DVB-St Resin

**Figure 5:**
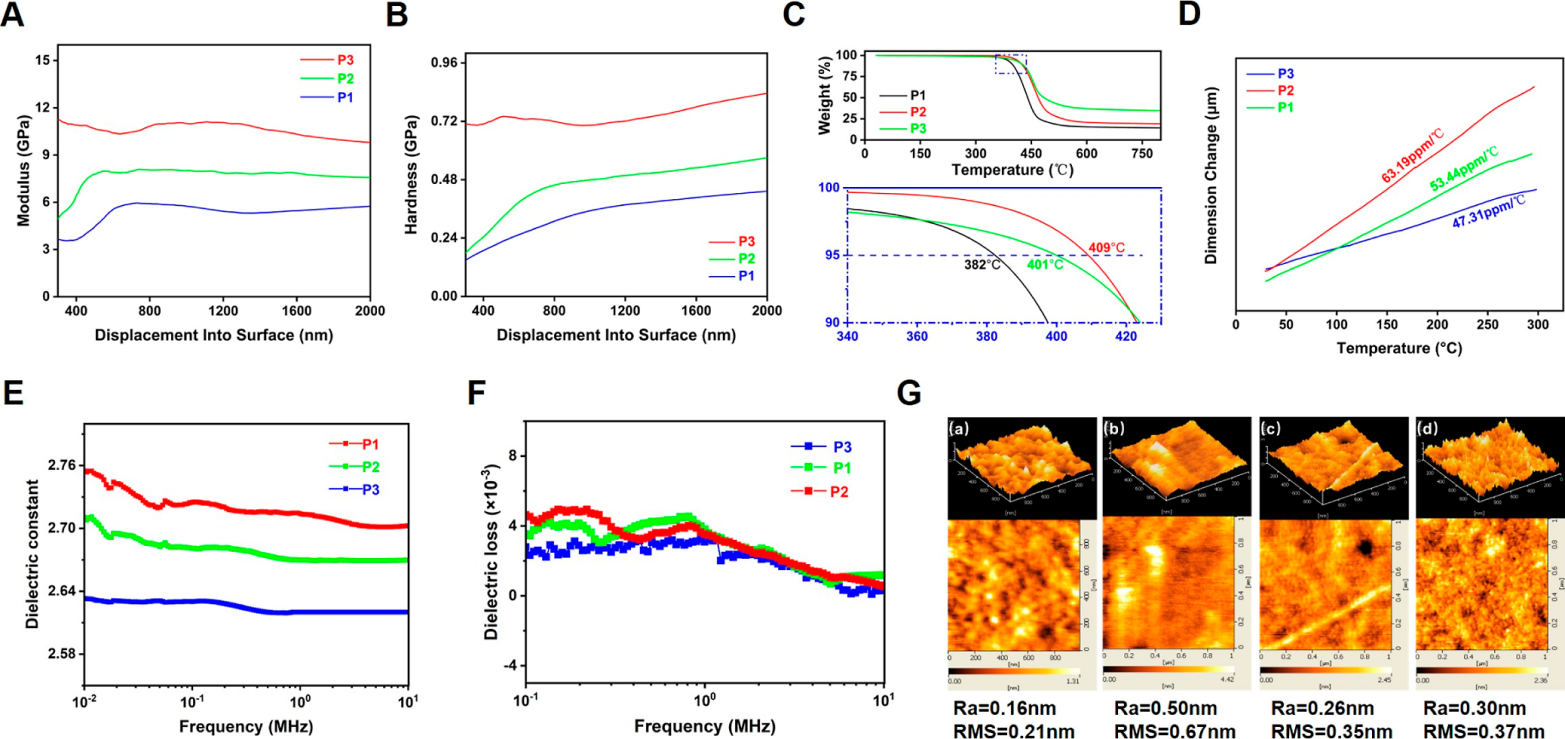
(A) Modulus of UV/thermal-cured P-DVB-St resin. (B) hardness of UV/thermal-cured P-DVB-St resin. (C) TG curves of UV/thermal-cured P-DVB-St resin. (D) CTE curves of UV/thermal-cured P-DVB-St resin. (E) Dielectric constants with the changing frequency of UV/thermal-cured P-DVB-St resin. (F) Dielectric loss with the changing frequency of UV/thermal-cured P-DVB-St resin. (G) AFM plots of photosensitive thin films: (a) Uncured film; (b) UV-cured film; (c) UV/thermal-cured films; and (d) direct heat-cured films.

In this paper, nanoindentation is used to characterize the mechanical properties of UV/thermal-cured resins. As shown in Figure 5A,B, P3 exhibits the highest modulus (10.5 GPa) and hardness (0.7 GPa) due to its elevated cross-linking density. Conversely, the mechanical properties of P2 and P1 diminish as the number of double bonds and the BCB group decline. The modulus and hardness of the polymers change drastically at less than 500 nm, probably due to the size effect of nanoindentation,^36,37^ which is affected by the cross-link density of the polymers, surface roughness, defects at the tip of the indenter, and so forth. The modulus and hardness fluctuate considerably, but the data tend to be stabilized with the increase of the depth of indentation.

The coefficient of linear thermal expansion (CTE) is one of the most critical characteristics in the semiconductor industry. Similarly, the resins demonstrate excellent thermal stability, as illustrated in Figure 5C. The *T*_5%_ temperature is 401 °C for P3, 409 °C for P2, and 382 °C for P1, and the polymers exhibit a minimal weight loss until 350 °C, which aligns with the annealing temperatures typically required for packaging materials in microelectronic ICs. Figure 5D demonstrates the CTE curves of the photo-/thermal-cured P-DVB-St. The CTE of P3, P2, and P1 are 47.31, 53.44, and 63.19 ppm/°C, respectively, indicating that the material has good resistance to temperature change. This is due to the fact that the thermal ring-opening polymerization of the BCB units improves the dimensional stability of the cured resin at high temperatures, and the more BCB structural units there are, the smaller the CTE. In conclusion, the P-DVB-St-cured resin exhibits excellent thermal stability properties, which are contingent upon the resin’s UV/thermal dual-cured structure.

Dielectric constant and dielectric loss are used as one of the core parameters of microelectronic interlayer packaging and are calculated by measuring the impedance at ambient temperature. Figure 5E,F shows the dielectric constant and dielectric loss of the three polymers at 0–10 MHz. The one with the lowest dielectric constant and dielectric loss is P3 with 2.62 and 1.7 × 10^–3^. According to the derivation of the Debye relaxation equation^38^
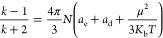
where *k* is the dielectric constant, *T* is the temperature, *N* is the number density of dipoles, *a*_e_ is the electrode polarization, *a*_d_ is the aberration polarization, μ is the orientation polarization associated with the dipole moment, and *K*_b_ is the Boltzmann constant. The low polarity of the all-hydrocarbon structure reduces the electrode polarization *a*_e_, while the high cross-linking density network structure formed by the UV/thermal dual-cured structure effectively prevents molecular buildup and distorted polarization, thereby reducing the aberration polarization *a*_d_ and dipole density *N*. The P-DVB-St resin is an amorphous polymer, and the smaller anisotropy reduces μ. The aforementioned factors result in reduced *k* values. The increase in the doping ratio of DVB in P-DVB-St enhances the dielectric constant of the cured resin. In this paper, the surface morphology of polymer films is analyzed by AFM. As shown in Figure 5G, (a) is an uncured photosensitive film with the average surface roughness (*R*_a_) and root-mean-square roughness (RMS) of 0.16 and 0.21 nm, respectively. *R*_a_ and RMS of the photocured film (b) are 0.5 and 0.67 nm, which may be attributed to the oxidization of the polymer film as well as the structural shrinkage after photocuring that leads to the increase of surface roughness. The UV-cured film was heat-cured again to obtain the UV/thermal-cured film (c), and *R*_a_ and RMS decreased to 0.26 and 0.35 nm, which may be attributed to the highly cross-linked structure inhibiting the interfacial inhomogeneity on the surface of the copolymers, thus reducing the roughness.^39^ In addition, the *R*_a_ and RMS values of 0.30 and 0.37 nm for the direct heat-cured film (*d*) were larger than those of the photothermally cured film. Therefore, UV curing prior to heat curing can effectively reduce the surface roughness.

The comprehensive properties of P-DVB-St resins are shown in Table 3. The properties of samples P1, P2, and P3 show clear trends related to the polymer structure.

**Table 3:**
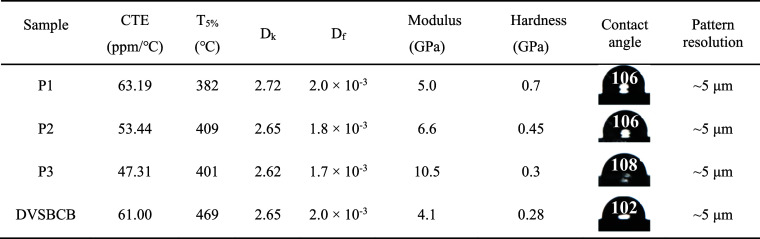
Comprehensive Properties of P-DVB-St Resin

The coefficient of thermal expansion (CTE) decreases from P1 to P3. This is because the increase in the DVB content leads to a higher cross-link density, enhancing the dimensional stability of the polymer and reducing the CTE. The thermal stability (*T*_5%_) generally increases with the rise in the DVB content. The more DVB units, the more rigid and stable the polymer structure under high temperatures. Both the dielectric constant (*D*_k_) and dielectric loss (*D*_f_) decrease as the DVB content increases. The all-hydrocarbon structure and high cross-linking density network structure resulting from more DVB units effectively reduce the polarization and dipole density, thus lowering *D*_k_ and *D*_f_. The modulus and hardness increase from P1 to P3. Higher DVB content means higher cross-linking density, which provides greater rigidity and strength to the polymer.

These variations in physical properties are directly associated with the changes in the polymer structure and cross-linking density as the ratio of DVB to St monomers is adjusted.

The developed P-DVB-St resins show superiority over DVSBCB in multiple aspects. In terms of thermal stability, P3 has *T*_5%_ of 401 °C, while DVSBCB has 469 °C. Although DVSBCB has a slightly higher *T*_5%_, the thermal stability of P-DVB-St resins is still excellent and can meet the requirements of most microelectronic applications. The dielectric properties of P3 are outstanding with a dielectric constant of 2.62 and a dielectric loss of 1.7 × 10^–3^, compared to DVSBCB’s 2.65 and 2.0 × 10^–3^, respectively. The lower values of P-DVB-St indicate better insulation performance between metal wires.

For mechanical properties, P3 has a modulus of 10.5 GPa and hardness of 0.7 GPa, much higher than DVSBCB’s 4.1 and 0.28 GPa. This means P-DVB-St resins can provide better mechanical support and durability. In patterning resolution, both can reach about 5 μm, but P-DVB-St resins can be synthesized through a simpler and more cost-effective anionic polymerization process, enabling better control over the polymer structure and molecular weight.

In conclusion, P-DVB-St resins have a comprehensive performance advantage and greater potential in microelectronic packaging applications such as in the redistribution layers of FO-WLP, where low dielectric constant, good mechanical properties, and controllable synthesis are highly desired.

## 4 Conclusions

In this study, a class of fully hydrocarbon benzocyclobutene film-forming resins with light/heat dual-curing structures with molecular weights in the range of 1–2 were synthesized by anionic polymerization, P-DVB-St. The polymer structure and properties were modulated by adjusting the copolymerization ratio (DVB: St) to meet different application scenarios. The P-DVB-St resins were compounded into photoresists with the preferred azide-cyclohexanone and coumarin photoinitiator systems. The P-DVB-St resin is compounded with the preferred azidocyclohexanone and coumarin photoinitiation systems to form a photoresist. The solution ratio and photolithography parameters are then precisely adjusted in order to achieve a precise pattern transfer (resolution of 5 μm and edge roughness of 0.5 μm). Subsequently, the photocured resin is thermally cured to yield a novel all-hydrocarbon low-dielectric resin exhibiting favorable thermal stability, dielectric properties, and mechanical characteristics.

As the feed ratio of the two monomers, DVB/St, increases, the thermal stability and mechanical properties of the P-DVB-St resin basically increase, while the dielectric constant tends to decrease. Among them, the P3 resin showed better thermal stability (*T*_5%_ = 401 °C), low dielectric constant and dielectric loss (*D*_k_ = 2.62, *D*_f_ = 1.7 × 10^–3^), low coefficient of thermal expansion (CTE = 47.31 ppm/°C), and high modulus and hardness (modulus = 10.5 GPa, hardness = 0.7 GPa) due to the higher cross-link density structure. The thermal stability, dielectrics, and mechanical properties of the resin can be tuned by varying the content of DVB structural units in the polymer. Compared to commercial DVSBCB,^40^ which requires thermal polymerization to achieve photosensitivity, P-DVB-St resin requires only a simple anionic polymerization reaction to achieve large-scale and precise control of the polymer structure and molecular weight at a relatively lower cost, making it a potentially commercialized high-performance encapsulation photoresist expected to be used in microelectronic packaging.

## Supporting Information Available

DTG curve of P-DVB-St resin and nano load–displacement curves of UV/thermal-cured P-DVB-St (PDF)

## Abbreviations

DVB: 1-(4-vinylphenyl)-2-(4-benzocyclobutenyl)ethene
St: styrene
BCB: benzocyclobutene
TEA: triethylamine
4-BrBCB: 4- bromobenzocyclobutene
DME: dipropylene glycol dimethyl ether
PE: petroleum ether
THF: tetrahydrofuran
NMR: nuclear magnetic resonance
FTIR: Fourier transform infrared
TGA: thermogravimetric analysis
DSC: differential scanning calorimetry
TMA: thermomechanical analysis
BAC: 2,6-bis(4-azidobenzylidene) cyclohexanone
*M*_w_: mass average molar mass
*M*_n_: number-average molar mass
PDI: polymer dispersity index
dye: 3,3′-carbonyl-bis[7-diethylaminocoumarin]
DTG: derivative thermogravimetry
CTE: coefficient of thermal expansion
UV: ultraviolet
*D*_k_: dielectric constant
*D*_f_: dielectric loss
ISC: intersystem crossing
