# Supplementary material for: All-Hydrocarbon Low-Dielectric Loss Benzocyclobutene-Encapsulated Photoresist with High Pattern Resolution
Source: ACS Omega. 2025 Apr 12;10(15):15219–28. doi: 10.1021/acsomega.4c10940 (PMC12019739; doi:10.1021/acsomega.4c10940)
Supplement: Supplementary file 1 — ao4c10940_si_001.pdf [file ao4c10940_si_001.pdf]

All-hydrocarbon low dielectric loss benzocyclobutene encapsulated photoresist with high pattern resolution

Hanlin Du,[a] Hongyan Xia,[b] Yun Tang,[a], c Ke Cao,[a] Jiajun Ma,[*a] and Junxiao Yang[*a]

a. School of Materials and Chemistry and State Key Laboratory of Environmentally-Friendly Energy Materials, Southwest University of Science and Technology, Mianyang 621010, China

b. Department of Applied Sciences, Northumbria University, Newcastle upon Tyne, NE1 8ST, UK

c. School of Materials and Construction, Mianyang Polytechnic, Mianyang 621000, China.

Content

Page2: The GPC spectrum of P-DVB-St **(Figure S1)**

Page2: Thickness of P-DVB-St photosensitive solution spin-coated film **(Figure S2)**

Page3: Purity of P-DVB-St full hydrocarbon resin **(Figure S3)**

Page3: Photolithography patterns for commercial DVSBCB photoresists **(Figure S4)**

Page4: In situ infrared of films irradiated by 150 mW/cm^2 light intensity ultraviolet **(Figure S5)**

Page5: UV-Vis Absorption Spectra of Photoresists **(Figure S6)**

Page7: Temperature ramp rheology test curve of P-DVB-St **(Figure S7)**

Page7: DTG curve of P-DVB-St resin **(Figure S8)**

Page8: Nano Load−Displacement curves of UV/Thermal cured P-DVB-St **(Figure S9)**

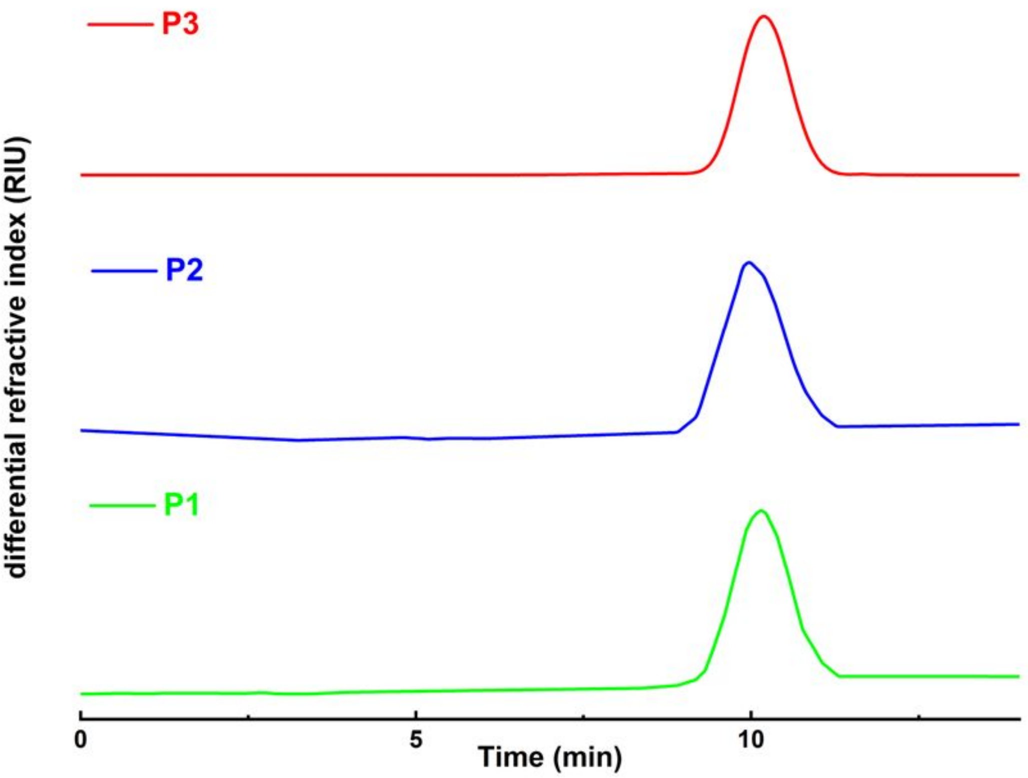

Figure S1 The GPC spectrum of P-DVB-St

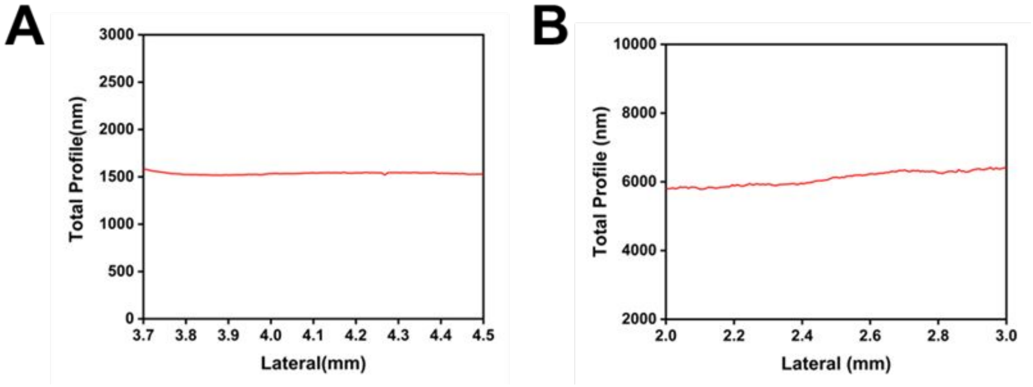

Figure S2 Thickness of P-DVB-St photosensitive solution spin-coated film: (A)1-2 μm；(B) 6-7 μm.

Test condition	
Combastion Furnace Temperature	1150 °C
Reducing Furnace Temperature	850 °C
Oxygen Flow Rate	15 mL/min
Helium Flow Rate	220 mL/min
Test Mode	CHNS, O
Linear Range	N:0.03~2 mg, C:0.03~2 mg, S:0.03~2 mg, H： 0.03~2 mg, O:0.03~2 mg
Analytic Precision	Standarddeviation ≤ 0.2%
Gas Purity	>99.999%

Results					
Sample	N(%)	C(%)	H(%)	S(%)	O(%)
1	0.00	90.73	7.89	0.00	0.00
1	0.00	90.23	7.85	0.00	0.00

Figure S3 Purity of P-DVB-St full hydrocarbon resin

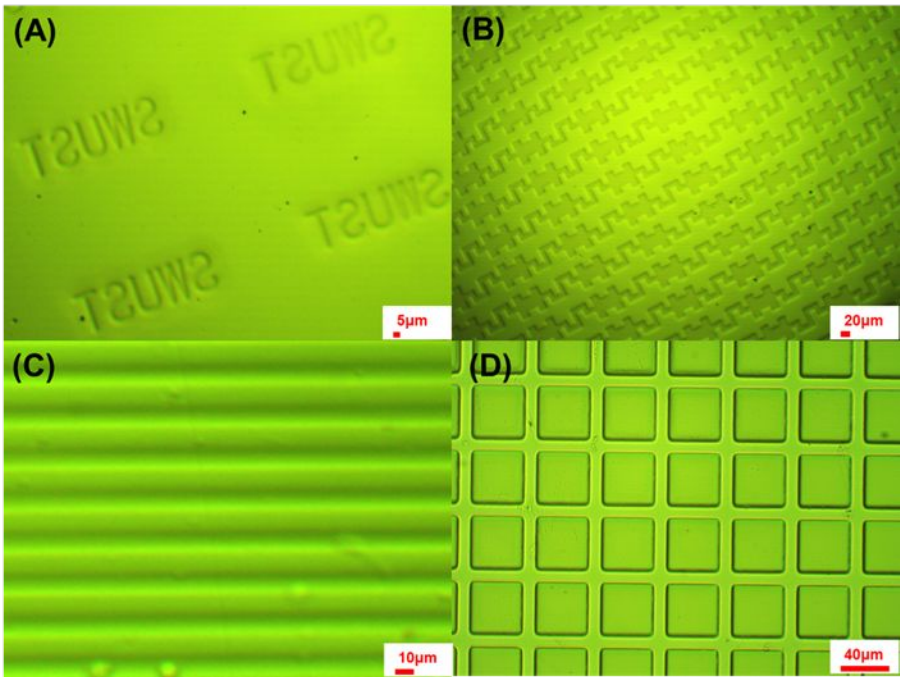

Figure S4 365-nm UV lithography pattern of commercial DVSBCB photoresist (A: letters with a line width of 5 μm; B: the patterned connecting line is a 20 μm sawtooth pattern; C: lines with 10 μm line width and spacing; D: a square with a side length of

40 μm)

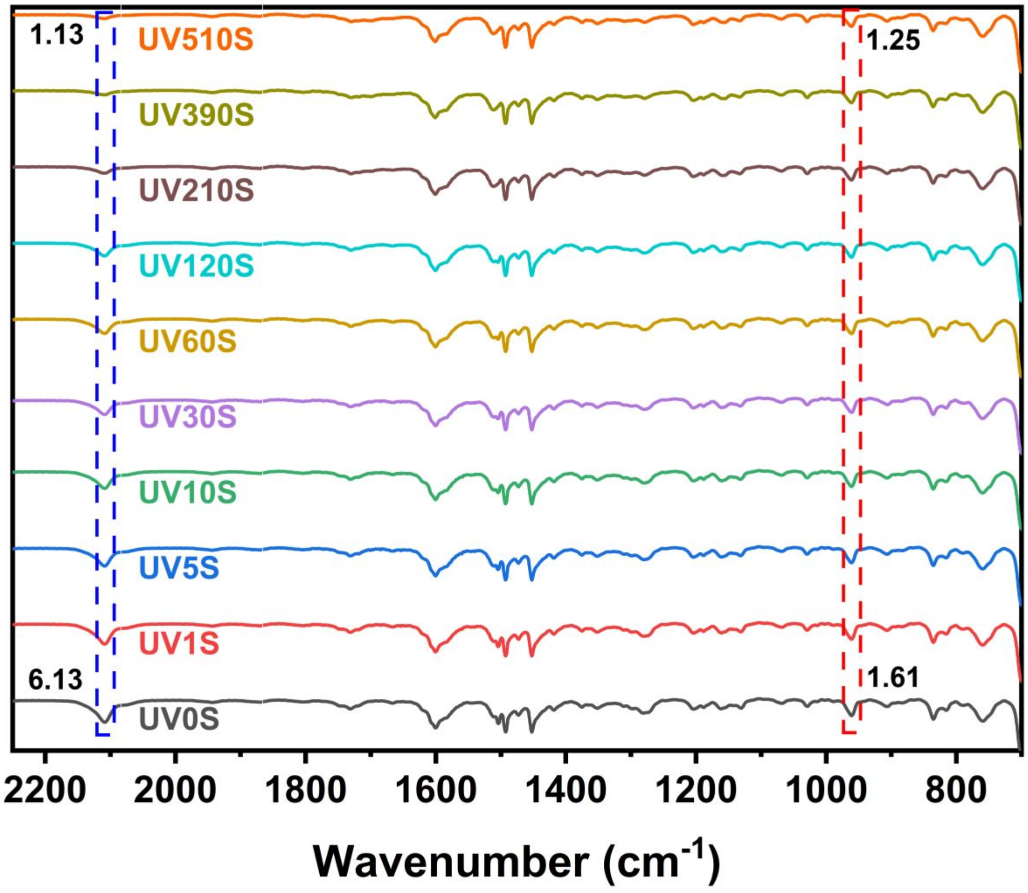

Figure S5 150 mW/cm^2 UV-cured kinetic FTIR spectrum of P-DVB-St photoresist

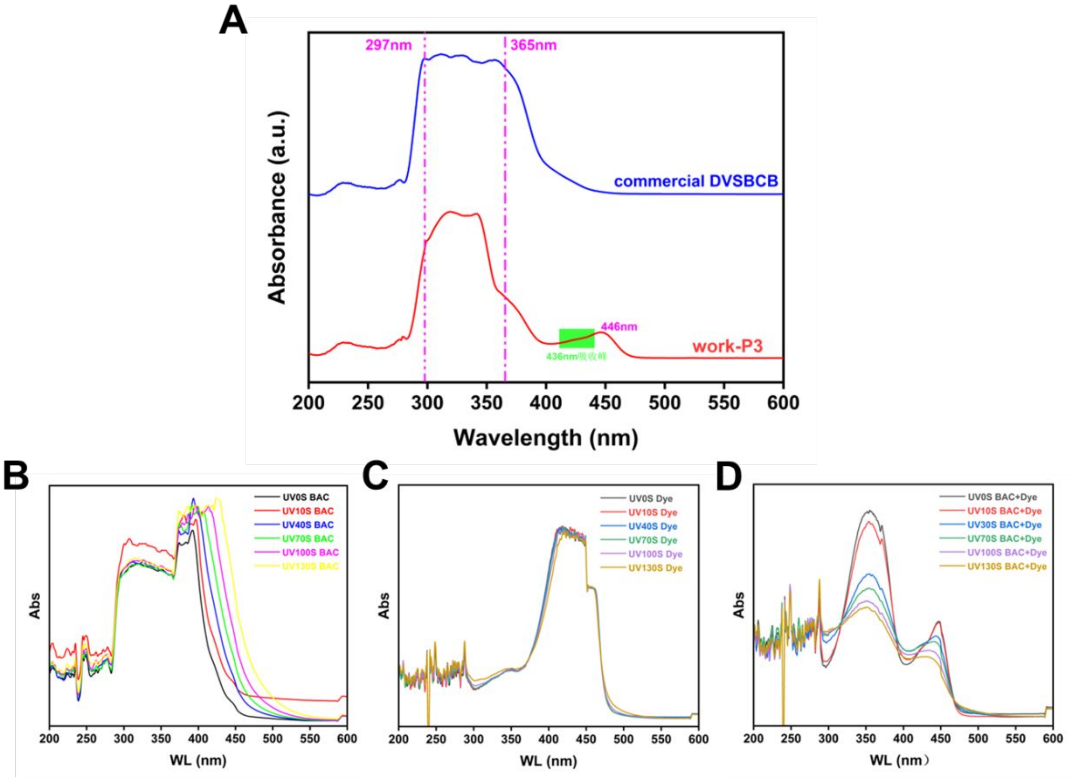

Figure S6 UV-Vis Absorption Spectra of Photoresists：（A）UV-Vis Absorption Spectra of P3 and commercial DVSBCB; (B) UV-Vis Absorption Spectra of BAC; (C) UV-Vis Absorption Spectra of Dye; (D) UV-Vis Absorption Spectra of BAC+Dye.

In this work, UV absorption experiments were carried out on the samples by UV- 4800 UV-visible spectrophotometer, and both P-DVB-St photoresist and commercial DVSBCB photoresist were used with homotrimethylbenzene as the solvent, and the UV absorption experiments were carried out under the same conditions. Because P3 photoresist has better light/heat curing performance, P3 photoresist and commercial DVSBCB photoresist were used for UV absorption comparison, see Figure S6A. Both photoresists have UV absorption starting at 280 nm, ending at 390 nm, and maximum absorption near 365 nm, so the study was carried out using 365-nm UV light for photolithography patterning studies to achieve maximum utilisation of energy. However, the P3 photoresist has a narrower absorption peak in the UV range, implying a faster photoresponse, higher photoactivity, and higher sample purity; moreover, the P3 sample still has UV absorption near 436 nm, indicating that the P3 photoresist has

higher wavelength selectivity. This is a UV absorption advantage determined by the structure of the matrix resin and the lithography system. In addition, the UV-VIS energy absorption properties of photoinitiated systems are also studied. The Figure S6B is the ultraviolet-visible absorption spectrum of the photoinitiator BAC. BAC has a characteristic maximum value around 350 nm, given by the π-π* transition in the aromatic ring next to the azide group, while the absorption peak gradually redshifts with the extension of the light time, which is caused by the Bac-BAC dimer connected by the azo bridge after the azide group cleavage[1]. The Figure S6C is the ultraviolet-visible absorption spectrum of the photoinitiator Dye. Due to the use of non-polar solvent tritylene, the UV absorption peaks of all coumarins are blue shifted. The ƛmax absorption peak is near 405 nm[2-3]. The Figure S6C shows the UV-VIS absorption spectrum of the photosensitive solution formed by the combination of BAC and Dye. In the complex system, coumarin absorbs wavelength energy into the excited state, and then transfers energy to the azide group by electron transfer reaction. This process of electron transfer causes the UV absorption peak to shift blue, so that the ƛmax absorption is shifted to 365nm, With the increase of illumination time, the absorption of BAC+Dye system at 365nm showed a gradient decline[4-6].

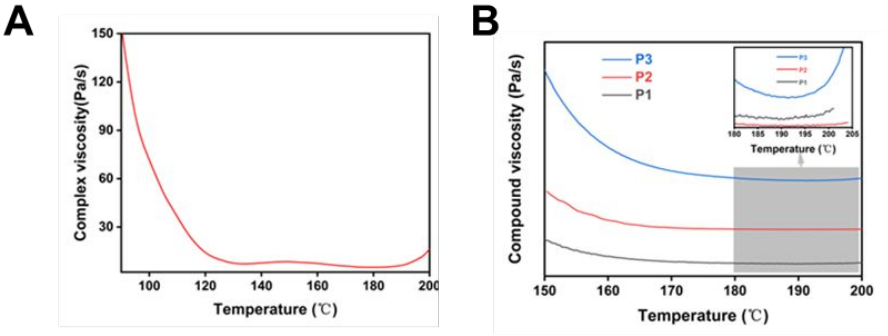

Figure S7 Temperature ramp rheology test curve of P-DVB-St; (A) P-DVB-St resin +10wt% DVB monomer; (B) Pure P-DVB-St resin

The P-DVB-St sample powder was spread evenly on the sample stage of the rheometer and the thickness of the sample was 1000 μm, which was measured by the programmed temperature increase under closed conditions. Figure S4A shows that the double bonds cross-link at 120 °C, resulting in an increase in the viscosity of the polymer, whereas the viscosity of the pure P-DVB-St resin in Figure S4B shows a decreasing trend up to 160 °C, indicating that no double bonds are involved in the cross-linking. The increase in viscosity at 180°C was greater than that caused by double bond polymerisation, suggesting a ring-opening polymerisation of BCB, while IR showed that the final polymer did not contain a double bond, demonstrating that double bond cross-linking and BCB curing occurred to the same extent.

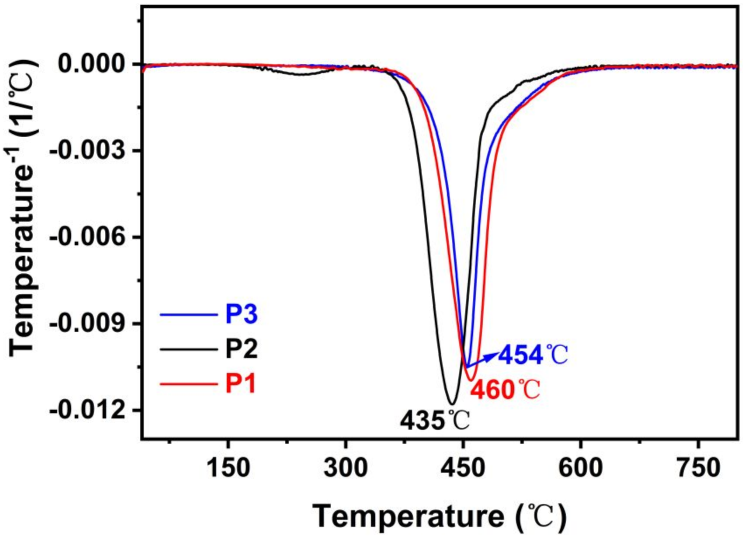

Figure S8 DTG curve of P-DVB-St resin, showing the decomposition temperature of the cured resin

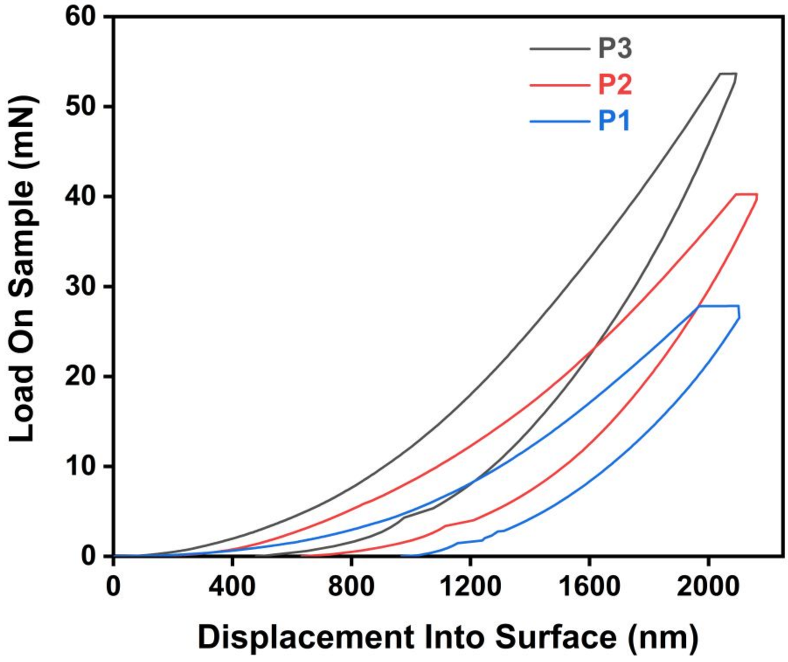

Figure S9 Nano Load−Displacement curves of UV/thermal cured P-DVB-St

Reference

(1) Avadanei, M. Photochemistry of 2, 6-di (4′-azidobenzylidene)-methylcyclohexanone in polymer matrices. Journal of Applied Polymer Science. 2017, 134(15). DOI: 10.1002/app.44694

(2) Nazir, R.; Danilevicius, P.; Ciuciu, A. I.; Chatzinikolaidou, M.; Gray, D.; Flamigni, L.; Farsari, M.; Gryko, D. T. π-expanded ketocoumarins as efficient, biocompatible initiators for two-photon-induced polymerization. Chemistry of Materials. 2014, 26(10): 3175-3184. DOI: 10.1021/cm500612w

(3) Dumur F. Recent advances on coumarin-based photoinitiators of polymerization. European Polymer Journal. 2022, 163: 110962. DOI: 10.1016/j.eurpolymj.2021.110962

(4) Fouassier, J. P.; Morlet-Savary, F.; Yamashita, K,; Imahashi, S. Visible light-induced polymerization reactions: The seven-role of the electron transfer process in the dye/iron arene complex/amine system. Journal of applied polymer science. 1996, 62(11): 1877-1885. DOI: 10.1002/(sici)1097-4628(19961212)62:11<1877::aid-app12>3.0.co;2-w

(5) Allonas, X.; Fouassier, J. P.; Kaji, M.; Miyasaka, M. On the ability of coumarin derivatives to interact with photoinitiators. Journal of Photopolymer Science and Technology. 2000, 13(2): 237-241. DOI: 10.2494/photopolymer.13.237

(6) Zheng, Y. Q.; Yao, Z. F.; Lei, T.; Dou, J. H.; Yang, C. Y.; Zou, L.; Meng, X. Y.; Ma, W.; Wang, J. Y.; Pei, J. Unraveling the solution-state supramolecular structures of donor–acceptor polymers and their influence on solid-state morphology and charge-transport properties. Advanced Materials. 2017, 29(42): 1701072. DOI: 10.1002/adma.201701072
